# Supplementary material for: Atmospheric emissions of hexachlorobutadiene in fine particulate matter from industrial sources
Source: Nat Commun. 2024 Jun 4;15:4737. doi: 10.1038/s41467-024-49097-0 (PMC11150375; doi:10.1038/s41467-024-49097-0)
Supplement: Supplementary file 1 — Supplementary Information [file 41467_2024_49097_MOESM1_ESM.pdf]

Supplementary Information for

Atmospheric emissions of hexachlorobutadiene in fine particulate matter from industrial sources

Chenyan Zhao[1,2,3], Lili Yang[1,3,*], Yuxiang Sun[1,2,3], Changzhi Chen[1,2,3], Zichun Huang[1,2,3], Qiuting Yang[1,3], Jianghui Yun[1,3], Ahsan Habib[4], Guorui Liu[1,2,3,*], Minghui Zheng[1,2,3], Guibin Jiang[1,2,3]

[1] State Key Laboratory of Environmental Chemistry and Ecotoxicology, Research Center for Eco-Environmental Sciences, Chinese Academy of Sciences, Beijing, 100085, China.

[2] School of Environment, Hangzhou Institute for Advanced Study, UCAS, Hangzhou, 310024, China.

[3] College of Resources and Environment, University of Chinese Academy of Sciences, Beijing, 100049, China.

[4] Department of Chemistry, Dhaka University, Dhaka 1000, Bangladesh

[*] Corresponding authors: llyang@rcees.ac.cn (L. Yang); grliu@rcees.ac.cn (G. Liu)

This document includes

Supplementary Table 1 Hexachlorobutadiene concentrations in products from different chemical industries.

Supplementary Table 2 Detail information on collected samples in this study.

Supplementary Table 3 Carcinogenic Risks (CR) of hexachlorobutadiene in various regions with 12 industries.

Supplementary Table 4 Carcinogenic risks (CR) of hexachlorobutadiene in various countries with 12 industries.

Supplementary Table 5 Methods for hexachlorobutadiene determination in solid sample and its concentrations.

Supplementary Table 6 Emission factors (EFs) of particulate matter (PM) and hexachlorobutadiene from different sources.

Supplementary Table 7 Annual production yield data sources for 12 industries.

Supplementary Figure 1 Atmospheric emissions of hexachlorobutadiene (HCBD) in particulate matter from industry on a provincial scale.

Supplementary Figure 2 The flowchart of this study on hexachlorobutadiene (HCBD) emissions and cancer risk evaluation.

Supplementary Figure 3 The scheme of geographic locations of investigated industrial plants.

Supplementary Figure 4 Gas chromatographic mass spectrometry spectra of hexachlorobutadiene (HCBD) and its isotopic internal standard.

Supplementary Table 1 Hexachlorobutadiene concentrations in products from different chemical industries.

CTC, Carbon Tetrachloride; HCCP, Hexachlorocyclopentadiene; PCE, Perchloroethylene; TCE, Trichloroethylene; UNEP, United Nations Environment Programme.

Items	Process	Concentration (ng/mL)	Reference	Year
CTC	Methane method	5.00×10^{-2}	UNEP, 2013	1991[1]
	Methane method (optimized)	5.00×10^{-3}	UNEP, 2013	1991[1]
	Methanol method	8.17×10^{-5}	Zhang, 2015	2015[2]
	Methanol method	3.96×10^{-7}	Wang, 2021	2021[3]
HCCP	Cyclopentadiene method	1.11×10^{-2}	UNEP, 2013	1991[1]
PCE	Acetylene method	4.00×10^{-3}	Beijing Normal University, 2014	2014
	CTC method	4.20×10^{-3}	Beijing Normal University, 2014	2014
	Acetylene method	7.60×10^{-8}	Wang, 2021	2021[3]
	CTC method	3.96×10^{-4}	Wang, 2021	2021[3]
TCE	Acetylene method	4.00×10^{-3}	UNEP, 2013	1991[1]
	Acetylene method	7.60×10^{-8}	Wang, 2021	2021[3]

Supplementary Table 2 Detail information on collected samples in this study.

CFPP, Coal-Fired Power Plant; CK, Cement Kilns; COP, Coke Production; EAF, Electric Arc Furnace Steelmaking; HWI, Hazardous Waste Incinerator; IOS, Iron Ore Sintering; MWI, Municipal Solid Waste Incineration; PCu, Primary Copper Smelting; SAl, Secondary Aluminum Smelting; SCu, Secondary Copper Smelting; SPb, Secondary Lead Smelting; SZn, Secondary Zinc Smelting.

Number	Sample ID	Concentration (ng/g)	Industrial activity	Raw material	Sample style	Average emission factors of hexachlorobutadiene (mg/kt·production)
1	CFPP-1	3.86	Coal-fired power plant	Bituminous coal, anthracite and lignite	Particulate matter	0.05
2	CFPP-2	2.13	Coal-fired power plant	Bituminous coal, anthracite and lignite	Particulate matter	
3	CFPP-3	1.47	Coal-fired power plant	Bituminous coal, anthracite and lignite	Particulate matter	
4	CFPP-4	7.48	Coal-fired power plant	Bituminous coal, anthracite and lignite	Particulate matter	
5	CFPP-5	n.d.	Coal-fired power plant	Bituminous coal, anthracite and lignite	Particulate matter	
6	CFPP-6	6.14	Coal-fired power plant	Bituminous coal, anthracite and lignite	Particulate matter	
7	CFPP-7	0.14	Coal-fired power plant	Bituminous coal, anthracite and lignite	Particulate matter	
8	CFPP-8	1.81	Coal-fired power plant	Bituminous coal, anthracite and lignite	Particulate matter	
9	CFPP-9	n.d.	Coal-fired power plant	Bituminous coal, anthracite and lignite	Particulate matter	
10	CFPP-10	11.92	Coal-fired power plant	Bituminous coal, anthracite and lignite	Particulate matter	
11	CFPP-11	1.42	Coal-fired power plant	Bituminous coal, anthracite and lignite	Particulate matter	

12	CFPP-12	7.48	Coal-fired power plant	Bituminous coal, anthracite and lignite	Particulate matter	
13	CFPP-13	46.25	Coal-fired power plant	Bituminous coal, anthracite and lignite	Particulate matter	
14	CFPP-14	3.05	Coal-fired power plant	Bituminous coal, anthracite and lignite	Particulate matter	
15	CFPP-15	6.71	Coal-fired power plant	Bituminous coal, anthracite and lignite	Particulate matter	
16	CK-1	0.60	Co-processing in cement kilns	Hazardous waste	Particulate matter	0.14
17	CK-2	1.03	Co-processing in cement kilns	Hazardous waste	Particulate matter	
18	CK-3	2.23	Co-processing in cement kilns	Hazardous waste	Particulate matter	
19	CK-4	1.06	Co-processing in cement kilns	Hazardous waste	Particulate matter	
20	CK-5	9.96	Co-processing in cement kilns	Hazardous waste	Particulate matter	
21	CK-6	2.94	Co-processing in cement kilns	Hazardous waste	Particulate matter	
22	COP-1	3.45	Coke production	Coke	Particulate matter	2.46
23	COP-2	14.28	Coke production	Coke	Particulate matter	
24	COP-3	19.03	Coke production	Coke	Particulate matter	
25	COP-4	13.14	Coke production	Coke	Particulate matter	
26	COP-5	n.d.	Coke production	Coke	Particulate matter	
27	COP-6	2.63	Coke production	Coke	Particulate matter	
28	COP-7	1.32	Coke production	Coke	Particulate matter	
29	COP-8	n.d.	Coke production	Coke	Particulate matter	
30	COP-9	0.62	Coke production	Coke	Particulate matter	
31	COP-10	13.12	Coke production	Coke	Particulate matter	
32	COP-11	2.49	Coke production	Coke	Particulate matter	

33	COP-12	1.63	Coke production	Coke	Particulate matter	
34	COP-13	0.56	Coke production	Coke	Particulate matter	
35	EAF-1	27.79	Electric arc furnace steelmaking	Scrap	Particulate matter	3.91
36	EAF-2	441.55	Electric arc furnace steelmaking	Scrap	Particulate matter	
37	EAF-3	18.01	Electric arc furnace steelmaking	Scrap	Particulate matter	
38	EAF-4	0.31	Electric arc furnace steelmaking	Scrap	Particulate matter	
39	EAF-5	1.49	Electric arc furnace steelmaking	Scrap	Particulate matter	
40	EAF-6	0.91	Electric arc furnace steelmaking	Scrap	Particulate matter	
41	EAF-7	11.14	Electric arc furnace steelmaking	Scrap	Particulate matter	
42	EAF-8	1.13	Electric arc furnace steelmaking	Scrap	Particulate matter	
43	EAF-9	n.d.	Electric arc furnace steelmaking	Scrap	Particulate matter	
44	EAF-10	n.d.	Electric arc furnace	Scrap	Particulate matter	

			steelmaking			
45	EAF-11	12.16	Electric arc furnace steelmaking	Scrap	Particulate matter	
46	EAF-12	5.37	Electric arc furnace steelmaking	Scrap	Particulate matter	
47	EAF-13	0.19	Electric arc furnace steelmaking	Scrap	Particulate matter	
48	EAF-14	0.70	Electric arc furnace steelmaking	Scrap	Particulate matter	
49	HWI-1	0.54	Hazardous waste incinerator	Hazardous waste such as medical waste	Particulate matter	0.23
50	HWI-2	1.07	Hazardous waste incinerator	Hazardous waste such as medical waste	Particulate matter	
51	HWI-3	0.27	Hazardous waste incinerator	Hazardous waste such as medical waste	Particulate matter	
52	HWI-4	39.93	Hazardous waste incinerator	Hazardous waste such as medical waste	Particulate matter	
53	HWI-5	0.21	Hazardous waste incinerator	Hazardous waste such as medical waste	Particulate matter	
54	HWI-6	47.40	Hazardous waste incinerator	Hazardous waste such as medical waste	Particulate matter	

55	HWI-7	1.30	Hazardous waste incinerator	Hazardous waste such as medical waste	Particulate matter	
56	HWI-8	0.73	Hazardous waste incinerator	Hazardous waste such as medical waste	Particulate matter	
57	HWI-9	0.95	Hazardous waste incinerator	Hazardous waste such as medical waste	Particulate matter	
58	HWI-10	0.10	Hazardous waste incinerator	Hazardous waste such as medical waste	Particulate matter	
59	HWI-11	176.56	Hazardous waste incinerator	Hazardous waste such as medical waste	Particulate matter	
60	HWI-12	27.05	Hazardous waste incinerator	Hazardous waste such as medical waste	Particulate matter	
61	HWI-13	0.65	Hazardous waste incinerator	Hazardous waste such as medical waste	Particulate matter	
62	HWI-14	28.83	Hazardous waste incinerator	Hazardous waste such as medical waste	Particulate matter	
63	IOS-1	n.d.	Iron ore sintering	Iron ore	Particulate matter	0.42
64	IOS-2	n.d.	Iron ore sintering	Iron ore	Particulate matter	
65	IOS-3	n.d.	Iron ore sintering	Iron ore	Particulate matter	
66	IOS-4	17.26	Iron ore sintering	Iron ore	Particulate matter	
67	IOS-6	1.88	Iron ore sintering	Iron ore	Particulate matter	

68	IOS-7	13.67	Iron ore sintering	Iron ore	Particulate matter	
69	IOS-8	12.59	Iron ore sintering	Iron ore	Particulate matter	
70	MWI-1	0.00	Municipal solid waste incineration	Municipal solid waste	Particulate matter	0.03
71	MWI-2	0.82	Municipal solid waste incineration	Municipal solid waste	Particulate matter	
72	MWI-3	0.95	Municipal solid waste incineration	Municipal solid waste	Particulate matter	
73	MWI-4	10.62	Municipal solid waste incineration	Municipal solid waste	Particulate matter	
74	MWI-5	1.32	Municipal solid waste incineration	Municipal solid waste	Particulate matter	
75	MWI-6	0.53	Municipal solid waste incineration	Municipal solid waste	Particulate matter	
76	MWI-7	n.d.	Municipal solid waste incineration	Municipal solid waste	Particulate matter	
77	MWI-8	12.46	Municipal solid waste incineration	Municipal solid waste	Particulate matter	
78	MWI-9	n.d.	Municipal solid waste incineration	Municipal solid waste	Particulate matter	
79	MWI-10	n.d.	Municipal solid waste	Municipal solid waste	Particulate matter	

			incineration			
80	MWI-11	0.36	Municipal solid waste incineration	Municipal solid waste	Particulate matter	
81	PCu-1	n.d.	Primary copper smelting	Copper ore	Particulate matter	1.30
82	PCu-2	9.40	Primary copper smelting	Copper ore	Particulate matter	
83	PCu-3	1.95	Primary copper smelting	Copper ore	Particulate matter	
84	PCu-4	8.59	Primary copper smelting	Copper ore	Particulate matter	
85	PCu-5	n.d.	Primary copper smelting	Copper ore	Particulate matter	
86	PCu-6	8.26	Primary copper smelting	Copper ore	Particulate matter	
87	SAl-1	n.d.	Secondary aluminum smelting	Waste aluminum	Particulate matter	2.45
88	SAl-2	1.25	Secondary aluminum smelting	Waste aluminum	Particulate matter	
89	SAl-3	6.48	Secondary aluminum smelting	Waste aluminum	Particulate matter	
90	SAl-4	4.33	Secondary aluminum smelting	Waste aluminum	Particulate matter	
91	SAl-5	16.89	Secondary aluminum smelting	Waste aluminum	Particulate matter	
92	SAl-6	1.84	Secondary aluminum smelting	Waste aluminum	Particulate matter	
93	SCu-1	20.40	Secondary copper smelting	Waste mixed copper	Particulate matter	660.85
94	SCu-2	2.05	Secondary copper smelting	Waste mixed copper	Particulate matter	
95	SCu-3	18.40	Secondary copper smelting	Waste mixed copper	Particulate matter	
96	SCu-4	3.75	Secondary copper smelting	Waste mixed copper	Particulate matter	
97	SCu-5	10144.37	Secondary copper smelting	Waste mixed copper	Particulate matter	
98	SCu-6	3457.63	Secondary copper smelting	Waste mixed copper	Particulate matter	

99	SCu-7	73.82	Secondary copper smelting	Waste mixed copper	Particulate matter	
100	SCu-8	33.49	Secondary copper smelting	Waste mixed copper	Particulate matter	
101	SCu-9	36.18	Secondary copper smelting	Waste mixed copper	Particulate matter	
102	SCu-10	6.35	Secondary copper smelting	Waste mixed copper	Particulate matter	
103	SPb-1	0.35	Secondary lead smelting	Waste lead	Particulate matter	3.22
104	SPb-2	n.d.	Secondary lead smelting	Waste lead	Particulate matter	
105	SPb-3	0.33	Secondary lead smelting	Waste lead	Particulate matter	
106	SPb-4	17.52	Secondary lead smelting	Waste lead	Particulate matter	
107	SPb-5	1.29	Secondary lead smelting	Waste lead	Particulate matter	
108	SPb-6	16.14	Secondary lead smelting	Waste lead	Particulate matter	
109	SPb-7	0.48	Secondary lead smelting	Waste lead	Particulate matter	
110	SPb-8	0.69	Secondary lead smelting	Waste lead	Particulate matter	
111	SPb-9	11.09	Secondary lead smelting	Waste lead	Particulate matter	
112	SPb-10	2.37	Secondary lead smelting	Waste lead	Particulate matter	
113	SPb-11	1.86	Secondary lead smelting	Waste lead	Particulate matter	
114	SZn-1	5.49	Secondary zinc smelting	Waste mixed zinc	Particulate matter	1.05
115	SZn-2	2.08	Secondary zinc smelting	Waste mixed zinc	Particulate matter	
116	SZn-3	0.80	Secondary zinc smelting	Waste mixed zinc	Particulate matter	
117	SZn-4	1.68	Secondary zinc smelting	Waste mixed zinc	Particulate matter	
118	SZn-5	3.05	Secondary zinc smelting	Waste mixed zinc	Particulate matter	
119	SZn-6	3.57	Secondary zinc smelting	Waste mixed zinc	Particulate matter	

120	SZn-7	n.d.	Secondary zinc smelting	Waste mixed zinc	Particulate matter
121	SZn-8	n.d.	Secondary zinc smelting	Waste mixed zinc	Particulate matter

Supplementary Table 3 Carcinogenic Risks (CR) of hexachlorobutadiene in various regions with 12 industries.

Region	CR						
	Adult			Child			Total
	CR_0	CR_1	Increase in CR (%)	CR_0	CR_1	Increase in CR (%)	Increase in CR (%)
East Asia & Pacific	6E-07	2E-06	278	9E-07	3E-06	278	278
Europe & Central Asia	6E-07	1E-06	101	9E-07	2E-06	101	101
Latin America & Caribbean	6E-07	7E-07	34	9E-07	1E-06	34	34
Middle East & North Africa	6E-07	7E-07	33	9E-07	1E-06	33	33
North America	6E-07	1E-06	118	9E-07	2E-06	118	118
South Asia	6E-07	2E-06	296	9E-07	3E-06	296	296
Sub-Saharan Africa	6E-07	6E-07	10	9E-07	1E-06	10	10

Supplementary Table 4 Carcinogenic risks (CR) of hexachlorobutadiene in various countries with 12 industries.

Country	Increase in hexachlorobutadiene concentration (mg/m³)	CR					
		Adult			Child		
		CR_0	CR_1	Increase in CR (%)	CR_0	CR_1	Increase in CR (%)
Algeria	4E-07	6E-07	6E-07	1%	9E-07	9E-07	1%
Andorra	1E-06	6E-07	6E-07	2%	9E-07	9E-07	2%
Argentina	9E-06	6E-07	6E-07	15%	9E-07	9E-07	15%
Australia	3E-06	6E-07	6E-07	4%	9E-07	9E-07	4%
Austria	1E-03	6E-07	1E-05	1990%	9E-07	9E-07	1990%
Azerbaijan	3E-06	6E-07	6E-07	5%	9E-07	9E-07	5%
Bahrain	1E-03	6E-07	1E-05	1911%	9E-07	9E-07	1911%
Bangladesh	5E-05	6E-07	1E-06	82%	9E-07	9E-07	82%
Belarus	2E-05	6E-07	7E-07	27%	9E-07	9E-07	27%
Belgium	5E-03	6E-07	4E-05	7930%	9E-07	9E-07	7930%
Bermuda	3E-05	6E-07	8E-07	43%	9E-07	9E-07	43%
Bolivia	1E-08	6E-07	6E-07	0%	9E-07	9E-07	0%
Bosnia and Herzegovina	6E-08	6E-07	6E-07	0%	9E-07	9E-07	0%
Botswana	3E-09	6E-07	6E-07	0%	9E-07	9E-07	0%
Brazil	8E-06	6E-07	6E-07	14%	9E-07	9E-07	14%
Bulgaria	2E-04	6E-07	3E-06	364%	9E-07	9E-07	364%
Cameroon	5E-09	6E-07	6E-07	0%	9E-07	9E-07	0%
Canada	7E-06	6E-07	6E-07	11%	9E-07	9E-07	11%

Chile	4E-06		6E-07	6E-07	6%		9E-07	9E-07		6%
China	3E-04		6E-07	4E-06	564%		9E-07	9E-07		564%
Colombia	2E-06		6E-07	6E-07	3%		9E-07	9E-07		3%
Croatia	2E-06		6E-07	6E-07	3%		9E-07	9E-07		3%
Cuba	3E-06		6E-07	6E-07	5%		9E-07	9E-07		5%
Denmark	2E-06		6E-07	6E-07	3%		9E-07	9E-07		3%
Dominican Republic	3E-07		6E-07	6E-07	0%		9E-07	9E-07		0%
Ecuador	3E-06		6E-07	6E-07	5%		9E-07	9E-07		5%
El Salvador	6E-06		6E-07	6E-07	10%		9E-07	9E-07		10%
Eritrea	2E-07		6E-07	6E-07	0%		9E-07	9E-07		0%
Estonia	2E-07		6E-07	6E-07	0%		9E-07	9E-07		0%
Finland	1E-04		6E-07	1E-06	163%		9E-07	9E-07		163%
France	4E-05		6E-07	9E-07	69%		9E-07	9E-07		69%
Germany	9E-04		6E-07	8E-06	1432%		9E-07	9E-07		1432%
Ghana	1E-07		6E-07	6E-07	0%		9E-07	9E-07		0%
Greece	1E-05		6E-07	7E-07	22%		9E-07	9E-07		22%
Guatemala	4E-06		6E-07	6E-07	6%		9E-07	9E-07		6%
Hungary	5E-06		6E-07	6E-07	8%		9E-07	9E-07		8%
Iceland	3E-07		6E-07	6E-07	0%		9E-07	9E-07		0%
India	2E-04		6E-07	3E-06	380%		9E-07	9E-07		380%
Indonesia	3E-05		6E-07	8E-07	44%		9E-07	9E-07		44%
Ireland	2E-07		6E-07	6E-07	0%		9E-07	9E-07		0%
Israel	4E-03		6E-07	4E-05	6905%		9E-07	9E-07		6905%

Italy	3E-04	6E-07	4E-06	574%	9E-07	9E-07	574%
Japan	1E-03	6E-07	1E-05	2299%	9E-07	9E-07	2299%
Jordan	5E-06	6E-07	6E-07	8%	9E-07	9E-07	8%
Kazakhstan	2E-06	6E-07	6E-07	4%	9E-07	9E-07	4%
Kenya	4E-08	6E-07	6E-07	0%	9E-07	9E-07	0%
Kuwait	9E-05	6E-07	1E-06	148%	9E-07	9E-07	148%
Libya	4E-07	6E-07	6E-07	1%	9E-07	9E-07	1%
Lithuania	6E-08	6E-07	6E-07	0%	9E-07	9E-07	0%
Malaysia	1E-04	6E-07	2E-06	183%	9E-07	9E-07	183%
Maldives	1E-06	6E-07	6E-07	2%	9E-07	9E-07	2%
Malta	1E-07	6E-07	6E-07	0%	9E-07	9E-07	0%
Mauritania	3E-08	6E-07	6E-07	0%	9E-07	9E-07	0%
Mexico	1E-04	6E-07	2E-06	235%	9E-07	9E-07	235%
Moldova	1E-05	6E-07	7E-07	25%	9E-07	9E-07	25%
Monaco	5E-04	6E-07	5E-06	776%	9E-07	9E-07	776%
Mongolia	5E-08	6E-07	6E-07	0%	9E-07	9E-07	0%
Montenegro	7E-06	6E-07	6E-07	12%	9E-07	9E-07	12%
Morocco	1E-06	6E-07	6E-07	2%	9E-07	9E-07	2%
Mozambique	2E-08	6E-07	6E-07	0%	9E-07	9E-07	0%
Myanmar	1E-06	6E-07	6E-07	2%	9E-07	9E-07	2%
Namibia	2E-07	6E-07	6E-07	0%	9E-07	9E-07	0%
Netherlands	5E-05	6E-07	1E-06	77%	9E-07	9E-07	77%
New Zealand	1E-06	6E-07	6E-07	2%	9E-07	9E-07	2%

Nigeria	1E-07	6E-07	6E-07	0%	9E-07	9E-07	0%
Norway	7E-07	6E-07	6E-07	1%	9E-07	9E-07	1%
Oman	3E-06	6E-07	6E-07	4%	9E-07	9E-07	4%
Pakistan	3E-06	6E-07	6E-07	5%	9E-07	9E-07	5%
Paraguay	1E-05	6E-07	6E-07	17%	9E-07	9E-07	17%
Peru	2E-06	6E-07	6E-07	3%	9E-07	9E-07	3%
Philippines	9E-06	6E-07	6E-07	15%	9E-07	9E-07	15%
Poland	4E-04	6E-07	4E-06	642%	9E-07	9E-07	642%
Portugal	3E-05	6E-07	8E-07	47%	9E-07	9E-07	47%
Qatar	3E-04	6E-07	3E-06	467%	9E-07	9E-07	467%
Republic of Korea	2E-03	6E-07	2E-05	2855%	9E-07	9E-07	2855%
Romania	6E-06	6E-07	6E-07	10%	9E-07	9E-07	10%
Russian Federation	2E-05	6E-07	8E-07	38%	9E-07	9E-07	38%
Saudi Arabia	2E-05	6E-07	8E-07	38%	9E-07	9E-07	38%
Serbia	1E-05	6E-07	7E-07	19%	9E-07	9E-07	19%
Singapore	1E-03	6E-07	1E-05	2263%	9E-07	9E-07	2263%
Slovenia	4E-05	6E-07	9E-07	65%	9E-07	9E-07	65%
South Africa	3E-05	6E-07	8E-07	43%	9E-07	9E-07	43%
Spain	2E-04	6E-07	2E-06	346%	9E-07	9E-07	346%
Sweden	1E-04	6E-07	2E-06	229%	9E-07	9E-07	229%
Switzerland	5E-05	6E-07	1E-06	82%	9E-07	9E-07	82%
Syrian Arab Republic	3E-08	6E-07	6E-07	0%	9E-07	9E-07	0%

Tajikistan	2E-08	6E-07	6E-07	0%	9E-07	9E-07	0%
Tanzania	1E-08	6E-07	6E-07	0%	9E-07	9E-07	0%
Thailand	2E-05	6E-07	7E-07	27%	9E-07	9E-07	27%
Tunisia	4E-07	6E-07	6E-07	1%	9E-07	9E-07	1%
Uganda	1E-04	6E-07	2E-06	172%	9E-07	9E-07	172%
Ukraine	2E-05	6E-07	7E-07	26%	9E-07	9E-07	26%
United Arab Emirates	6E-05	6E-07	1E-06	99%	9E-07	9E-07	99%
United Kingdom	4E-05	6E-07	9E-07	61%	9E-07	9E-07	61%
United States	1E-04	6E-07	2E-06	220%	9E-07	9E-07	220%
Uruguay	4E-07	6E-07	6E-07	1%	9E-07	9E-07	1%
Uzbekistan	2E-04	6E-07	2E-06	330%	9E-07	9E-07	330%
Viet Nam	2E-04	6E-07	2E-06	289%	9E-07	9E-07	289%
Zambia	1E-06	6E-07	6E-07	2%	9E-07	9E-07	2%
Zimbabwe	3E-08	6E-07	6E-07	0%	9E-07	9E-07	0%

Supplementary Table 5 Methods for hexachlorobutadiene determination in solid sample and its concentrations.

UAE: ultrasonic-assisted extraction; DCM: dichloromethane; Hex: n-hexane; ASE: accelerated solvent extraction.

Sample	Concentration	Extraction method	Extraction solvent	Clean-up method	Reference
Indoor dust	-	UAE	Hex/Acetone	Florisil SPE	[4]
Fly ash in municipal solid waste	1.39-7.81 ng/g	UAE	DCM	Multilayer column: Florisil, silicagel, Na_2SO_4	[5]
Fly ash in industrial solid waste	43.8 ng/g	UAE	DCM	Multilayer column: Florisil, silicagel, Na_2SO_4	[5]
Fly ash in rotary kilns	0.25 ng/g	Soxhlet extraction	DCM	Multilayer column	[6]
Air-dried soil	-	ASE	DCM/Hex	Multilayer column: Florisil, silicagel, Na_2SO_4, activated copper	[7]
Freeze-dries soil	-	Soxhlet extraction	Hex/Acetone	3% water activated Florisil column	[8]

Supplementary Table 6 Emission factors (EFs) of particulate matter (PM) and hexachlorobutadiene from different sources.

SCu, Secondary Copper Smelting; COP, Coke Production; EAF, Electric Arc Furnace Steelmaking; IOS, Iron Ore Sintering; SAl, Secondary Aluminum Smelting; CFPP, Coal-Fired Power Plant; SPb, Secondary Lead Smelting; SZn, Secondary Zinc Smelting; CK, Cement Kilns; MWI, Municipal Solid Waste Incineration; HWI, Hazardous Waste Incinerator; CL, Coal Loading; CD, Coke Discharging; BFGH, Blast Furnace Gas Heating; DCQ, Dry Coke Quenching; WCQ, Wet Coke Quenching.

Industry	Raw Material	Product	Production Process	Production Scale	PM Producing Coefficient (kg/t)	PM removal equipment	PM removal efficiency	Final PM EFs (g/t)	EFs of hexachlorobutadiene for Estimation (kg/t)
SCu	Crude Copper	Anode Copper	Refining	-	0.07	Bag filter	0.990	1	0.479
	Low-grade Copper Scrap	Crude Copper	Fire Melting	-	31.07	Bag filter	0.980	621	
	How-grade Copper Scrap	Anode Copper	Pyro-refining	-	16.72	Bag filter	0.980	334	
	Anode Copper	Cathode Copper	Electrolyzation	-	-	Bag filter	-	-	
COP	Coking Coal	Coke	CL (Top-charging)	Oven Height > 6 m	-	Bag filter	0.102	102	0.442
			CD (Top-charging)		-	Bag filter	0.129	129	
			BFGH (Top-charging)		-	Bag filter	0.114	114	
			DCQ (Top-		-	Bag filter	0.105	105	

			charging)						
			CL (Top-charging)	6m > Oven Height > 4.3 m	-	Bag filter	0.121	121	
			CD (Top-charging)		-	Bag filter	0.134	134	
			BFGH (Top-charging)		-	Bag filter	0.119	119	
			DCQ (Top-charging)		-	Bag filter	0.113	113	
			CL (Top-charging)	4.3m > Oven Height	-	Bag filter	0.174	174	
			CD (Top-charging)		-	Bag filter	0.120	120	
			BFGH (Top-charging)		-	Bag filter	0.142	142	
			DCQ (Top-charging)		-	Bag filter	0.124	124	
			CL	All scales	-	Bag filter	0.115	115	
			CD		-	Bag filter	0.131	131	
			BFGH		-	Bag filter	0.120	120	
			WCQ		-	Bag filter	0.068	68	
EAF	Scrap Steel, Pig Iron, Iron Alloy, Slagging Agent	Carbon Steel	Electric Furnace Steelmaking	-	26.19	Bag filter	0.996	105	0.105
	Scrap Steel, Hot Iron, Ferrochrome, Direct Reduced Iron, Slagging	Alloy Steel		> 50 t	13.15	Bag filter	0.996	53	
				< 50 t	17.99	Bag filter	0.996	72	

	Agent								
	Scrap Steel, Hot Iron, Ferrochrome, Direct Reduced Iron, Slagging Agent	Stainless Steel		-	78.4	Bag filter	0.996	314	
IOS	Iron ore, Lime, Coke Powder, Pulverized Coal	Sinter	Sintering Machine Head	Sintering machines > 360 m^2	5.76	Bag filter	0.996	25	0.065
				180 m^2 < sintering machines < 360 m^2	5.81	Bag filter	0.996	25	
				sintering machines < 180 m^2	3.45	Bag filter	0.996	15	
			Sintering Machine Tail	sintering machines > 360 m^2	5.96	Bag filter	0.996	26	
				180 m^2 < sintering machines < 360 m^2	4.88	Bag filter	0.996	21	
				sintering machines < 180 m^2	3.6	Bag filter	0.996	15	
			Discharge	sintering machines >	6.54	Bag filter	0.996	28	

				360 m^2					
				180 m^2 < sintering machines < 360 m^2	5.6	Bag filter	0.996	24	
				sintering machines < 180 m^2	3.75	Bag filter	0.996	16	
SAl	Bauxite	Alumina	Bayer's method (95%)	All scales	13.86	Bag filter	0.995	0.0693	0.478
	Bauxite	Alumina	Hybrid method		23.85	Bag filter	0.995	0.11925	
	alumina	Primary aluminum	Molten salt electrolysis		40.88	Bag filter	0.990	0.4088	
CFPP*	Coal	Electricity/ electricity + heat	Pulverized coal boiler	450-749 megawatt	9.2Aar+9.33	Bag filter	0.999	0.00876	0.008
			Circulating fluidized bed boiler		5Aar+26.98Sar +7	Bag filter			
			Pulverized coal boiler	250-449 megawatt	9.21Aar+11.13	Bag filter		0.008165	
			Circulating fluidized bed boiler		6.31Aar+7.54+ 61.94Sar	Bag filter			
			Pulverized coal boiler	150-249 megawatt	9.33Aar+7.77	Bag filter		0.009335	
			Circulating fluidized bed boiler		6.24Aar+7.57+ 61.94Sar	Bag filter			

			Pulverized coal boiler	75-149 megawatt	9.31Aar+9.18	Bag filter		0.00767	
			Circulating fluidized bed boiler		6.31Aar+61.94Sar+7.27	Bag filter			
			Pulverized coal boiler	35-74 megawatt	9.36Aar+10.44	Bag filter		0.008225	
			Circulating fluidized bed boiler		6.24Aar+7.24+61.94Sar	Bag filter			
			Pulverized coal boiler	20-34 megawatt	9.16Aar+0.45	Bag filter		0.00884	
			Circulating fluidized bed boiler		6.3Aar+7.79+61.94Sar	Bag filter			
			Pulverized coal boiler	9-19 megawatt	9.18Aar+7.56	Bag filter		0.00412	
			Circulating fluidized bed boiler		6.3Aar+8.97+61.94Sar	Bag filter			
SPb	Lead concentrate + lead waste	Electrolytic lead	Flash lead smelting process	All scales	134.656	Bag filter-coated film	0.995	0.67328	0.680
	Lead bearing waste	Crude lead	Oxygen-rich melting furnace process		137.325		0.995	0.686625	
SZn	Zinc containing waste	Electro zinc	Fuming furnace - acid	All scales	50.201	Bag filter-coated	0.990	0.50201	0.502

			leaching - electrolysis process			film			
CK	Co-processing in cement kilns	Cement	Calcium, silicon aluminum, iron raw materials	All scales	94.57	Bag filter	0.995	0.05	0.05
MWI/HWI	Municipal solid waste incineration		Municipal solid waste	All scales					0.01

* When calculating the output, the electricity generated from thermal power generation was converted into standard coal consumption, with a conversion factor of 0.1228 kg of standard coal per 1 kWh of electric energy. This conversion allows for more precise and accurate results.

Supplementary Table 7 Annual production yield data sources for 12 industries.

CFPP, Coal-Fired Power Plant; CK, Cement Kilns; COP, Coke Production; EAF, Electric Arc Furnace Steelmaking; HWI, Hazardous Waste Incinerator; IOS, Iron Ore Sintering; MWI, Municipal Solid Waste Incineration; PCu, Primary Copper Smelting; SAl, Secondary Aluminum Smelting; SCu, Secondary Copper Smelting; SPb, Secondary Lead Smelting; SZn, Secondary Zinc Smelting.

	Website Name/Institution name	URL
SZn	U.S. Geological Survey.	https://www.usgs.gov/
SPb	U.S. Geological Survey.	https://www.usgs.gov/
SCu	International Copper Study Group	https://icsg.org/
PCu	International Copper Study Group	https://icsg.org/
SAl	International Aluminium institute.	https:// alucycle.world-aluminium.org/
HWI	The Word Bank.	https:// datacatalog.worldbank.org/
MWI	The Word Bank.	https:// datacatalog.worldbank.org/
EAF	World Steel Association.	https://www. worldsteel.org/
COP	The Word Bank.	https:// datacatalog.worldbank.org/
CK	U.S. Geological Survey.	https://www.usgs.gov/
CFPP	Energy Institute	https://www.energyinst.org/
IOS	World Steel Association.	https://www. worldsteel.org/

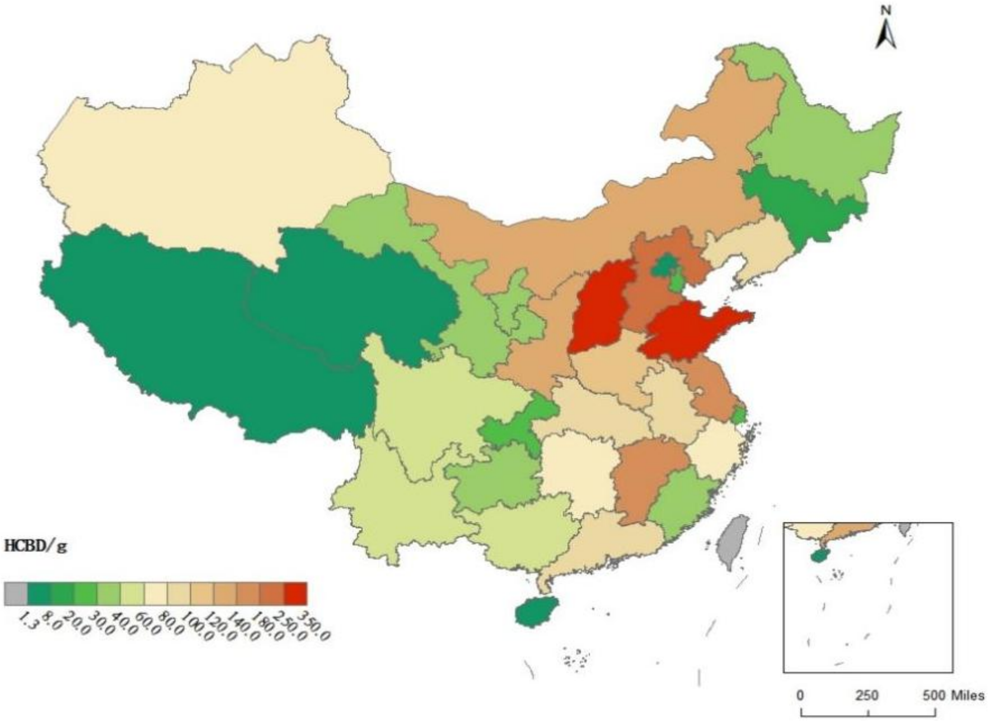

Supplementary Figure 1 Atmospheric emissions of hexachlorobutadiene (HCBD) in particulate matter from industry on a provincial scale.

We draw the map according to vector data sourced from “Resource and environmental science data registration and publication system [GS(2020)4619]” (https://www.resdc.cn/DOI/doi.aspx?DOIid=122) by Arcgis Pro software.

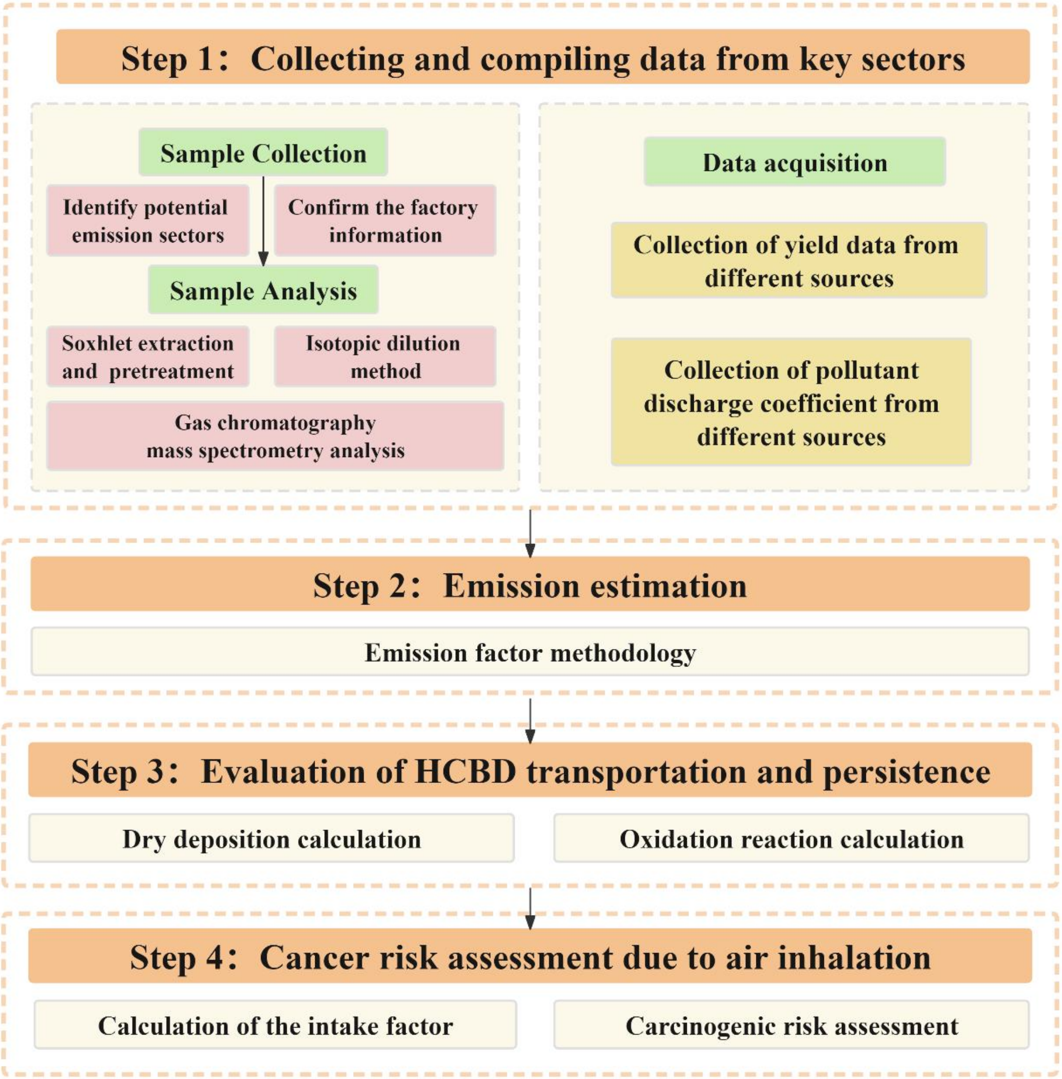

Supplementary Figure 2 The flowchart of this study on hexachlorobutadiene (HCBD) emissions and cancer risk evaluation.

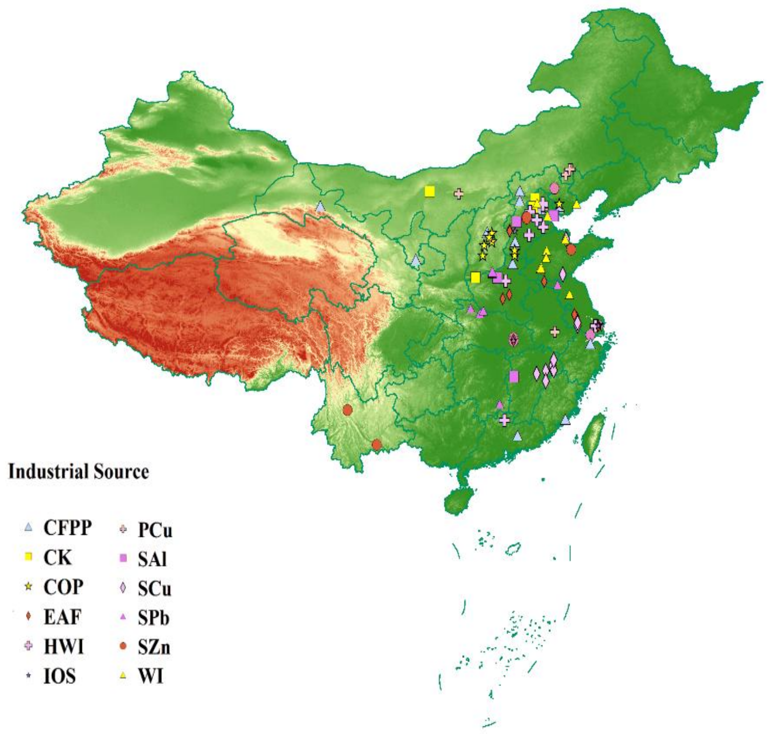

Supplementary Figure 3 The scheme of geographic locations of investigated industrial plants.

We draw the map according to vector data sourced from "Resource and environmental science data registration and publication system [GS(2020)4619]" (https://www.resdc.cn/DOI/doi.aspx?DOIid=122) by Arcgis Pro software.

CFPP, Coal-Fired Power Plant; CK, Cement Kilns; COP, Coke Production; EAF, Electric Arc Furnace Steelmaking; HWI, Hazardous Waste Incinerator; IOS, Iron Ore Sintering; MWI, Municipal Solid Waste Incineration; PCu, Primary Copper Smelting; SAl, Secondary Aluminum Smelting; SCu, Secondary Copper Smelting; SPb, Secondary Lead Smelting; SZn, Secondary Zinc Smelting.

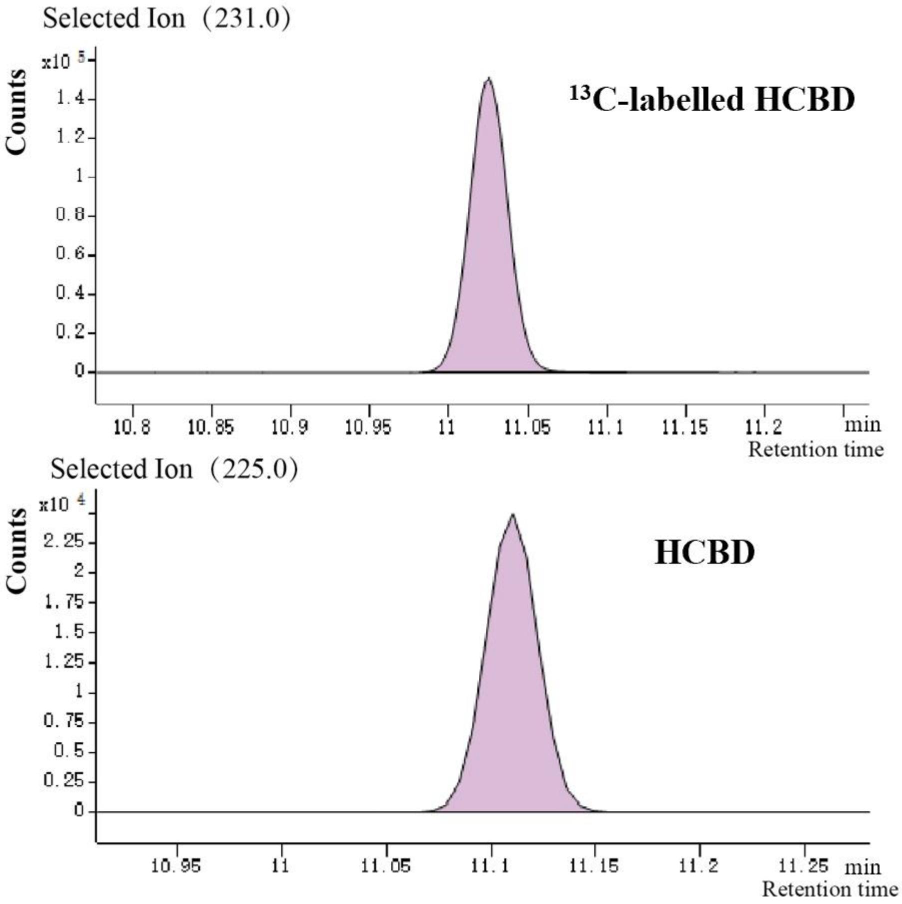

Supplementary Figure 4 Gas chromatographic mass spectrometry spectra of hexachlorobutadiene (HCBD) and its isotopic internal standard.

Supplementary References:

[1] UNEP. Risk management evaluation on hexachlorobutadiene. https://pops.int (2013).

[2] Zhang, L., Yang, W., Zhang, L. & Li, X. Highly chlorinated unintentionally produced persistent organic pollutants generated during the methanol-based production of chlorinated methanes: A case study in China. *Chemosphere* **133**, 1-5 (2015).

[3] Wang, M. et al. Hexachlorobutadiene emissions from typical chemical plants. *Front. Environ. Sci. Eng.* **15**, 60 (2021).

[4] van Drooge, B. L., Marco, E. & Grimalt, J. O. Atmospheric pattern of volatile organochlorine compounds and hexachlorobenzene in the surroundings of a chlor-alkali plant. *Sci. Total Environ.* **628–629,** 782–790 (2018).

[5] Zhang, H. et al. Determination of hexachlorobutadiene, pentachlorobenzene, and hexachlorobenzene in waste incineration fly ash using ultrasonic extraction followed by column cleanup and GC-MS analysis. *Anal. Bioanal. Chem.* **410**, 1893–1902 (2018).

[6] Kajiwara, N. et al. Environmentally sound destruction of hexachlorobutadiene during waste incineration in commercial- and pilot-scale rotary kilns. *J. Environ. Chem. Eng.* **7**, 103464 (2019).

[7] Zhang, H. et al. Levels and distributions of hexachlorobutadiene and three chlorobenzenes in biosolids from wastewater treatment plants and in soils within and surrounding a chemical plant in China. *Environ. Sci. Technol.* **48**, 1525–1531 (2014).

[8] Fang, Y. et al. Organochlorine pesticides in soil, air, and vegetation at and around a contaminated site in southwestern China: Concentration, transmission, and risk evaluation. *Chemosphere* **178**, 340–349 (2017).
